# HIV-1 Nef sequesters MHC-I intracellularly by targeting early stages of endocytosis and recycling

**DOI:** 10.1038/srep37021

**Published:** 2016-11-14

**Authors:** Brennan S. Dirk, Emily N. Pawlak, Aaron L. Johnson, Logan R. Van Nynatten, Rajesh A. Jacob, Bryan Heit, Jimmy D. Dikeakos

**Affiliations:** 1 Department of Microbiology and Immunology, The University of Western Ontario, Schulich School of Medicine and Dentistry, London, Ontario, Canada

## Abstract

A defining characteristic of HIV-1 infection is the ability of the virus to persist within the host. Specifically, MHC-I downregulation by the HIV-1 accessory protein Nef is of critical importance in preventing infected cells from cytotoxic T-cell mediated killing. Nef downregulates MHC-I by modulating the host membrane trafficking machinery, resulting in the endocytosis and eventual sequestration of MHC-I within the cell. In the current report, we utilized the intracellular protein-protein interaction reporter system, bimolecular fluorescence complementation (BiFC), in combination with super-resolution microscopy, to track the Nef/MHC-I interaction and determine its subcellular localization in cells. We demonstrate that this interaction occurs upon Nef binding the MHC-I cytoplasmic tail early during endocytosis in a Rab5-positive endosome. Disruption of early endosome regulation inhibited Nef-dependent MHC-I downregulation, demonstrating that Nef hijacks the early endosome to sequester MHC-I within the cell. Furthermore, super-resolution imaging identified that the Nef:MHC-I BiFC complex transits through both early and late endosomes before ultimately residing at the trans-Golgi network. Together we demonstrate the importance of the early stages of the endocytic network in the removal of MHC-I from the cell surface and its re-localization within the cell, which allows HIV-1 to optimally evade host immune responses.

The human immunodeficiency virus type 1 (HIV-1) encodes a class of proteins that lack any known enzymatic activity. These proteins, termed “accessory proteins”, include Nef, Vpr, Vpu and Vif. Accessory proteins can promote viral fitness by allowing infected cells to evade the host immune response^1^. The ability of Nef to promote HIV-1 immune evasion has been ascribed to its extensive interaction network with host proteins^2^,^3^,^4^. Indeed, Nef interacts with multiple proteins implicated in membrane trafficking in order to downregulate cell surface levels of major histocompatibility complex class I (MHC-I), resulting in a decreased ability of infected CD4^+^ T-cells to be detected and killed by CD8^+^ cytotoxic T lymphocytes (CTLs)^5^. This rerouting of MHC-I away from the cell surface is an example of a viral protein usurping host cell functions to ensure viral replication.

Currently, two models explain how Nef orchestrates the re-localization of MHC-I away from the cell surface (reviewed in ref. 6). The first model, termed the signaling mode of downregulation, is activated early during infection and involves the targeting of Nef to the trans-Golgi network (TGN) by the host membrane trafficking regulator protein phosphofurin acidic cluster sorting protein 2 (PACS-2)^2^,^7^. Once at the TGN, Nef binds and activates specific Src-family kinases (SFKs), which subsequently trigger the phosphoinositide 3-kinase (PI3K)-dependent endocytosis of cell surface MHC-I^3^. Internalized MHC-I is then sequestered in an intracellular compartment by a process involving the cytoplasmic tail of MHC-I^8^ and the membrane trafficking regulator phosphofurin acidic cluster sorting protein 1 (PACS-1), which has previously been identified to interact with the membrane adaptor protein-1 (AP-1)^2^,^3^,^9^. Interestingly, Nef, AP-1 and MHC-I have been described to form a ternary complex which depends on the cytoplasmic tail of MHC-I^10^. Structural information obtained by Jia *et al*. revealed that residues Y_320_ and D_327_ in the cytoplasmic tail of MHC-I bridge key interactions with Nef and AP-1, supporting Nef-dependent downregulation of MHC-I^10^.

The second model of Nef-dependent MHC-I downregulation, termed the stoichiometric mode, occurs at later stages of infection^7^. In this model, Nef interacts with the cytoplasmic tail of MHC-I and traffics the receptor to a degradative compartment in a process that also involves the membrane trafficking regulators AP-1 in addition to coat protein 1 (COPI)^11^. The signaling and stoichiometric models are not mutually exclusive and appear to be temporally linked^7^. Moreover, the signaling model may be more prominent in T-cells, as they have a relatively short half-life, whereas, the stoichiometric model may be more relevant in longer lived monocytes^7^.

Despite having identified multiple membrane trafficking regulator proteins implicated in the removal of MHC-I from the cell surface, it remains unknown what cellular compartments are used by Nef during this process precluding our understanding of the pathway subverted by HIV-1 to evade immune surveillance. In addition, prior analysis of pathways implicated in Nef-dependent MHC-I downregulation have primarily relied on the co-localization of Nef or MHC-I with markers of the membrane trafficking apparatus without analyzing Nef and MHC-I in complex, which is essential for Nef’s ability to downregulate MHC-I^12^,^13^. Importantly, the various compartments implicated in the trafficking of receptors such as MHC-I play distinct functional roles within the endosomal trafficking system^2^. Vesicles, such as early and late endosomes, are often implicated in the movement of cargo from the plasma membrane to distinct subcellular locations^14^,^15^. Moreover, late endosomes can also facilitate trafficking of cargo to degradative lysosomal compartments^16^. In parallel, recycling endosomes will continuously deplete proteins from the cell surface and return them to this location^17^. Fortunately the identity of the various intracellular compartments that comprise the endosomal trafficking system can be distinguished by specific effector molecules that coat the cytosolic face of these vesicles^18^.

We have previously localized interactions at the subcellular level between Nef and membrane trafficking regulators PACS-1 and PACS-2 using bimolecular fluorescence complementation (BiFC)^2^. This technique is used to study protein-protein interactions within cells and involves the reconstitution of a visible fluorophore from split fluorescent molecules expressed as fusion proteins on two distinct putative protein binding partners^19^,^20^,^21^. BiFC has enabled us and others to identify locations inside the cell where Nef can interact with itself or cytosolic binding partners, such as the trafficking regulator sorting nexin 18 (SNX18)^21^,^22^,^23^.

In the current report, we sought to determine the trafficking route undertaken by MHC-I in complex with Nef, in order to determine the fate of MHC-I in Nef-expressing cells. Using BiFC, we demonstrate that the Nef:MHC-I interaction is dependent on key residues in the cytoplasmic tail of MHC-I and we localize the Nef:MHC-I interaction within cells. Specifically, we show that Nef interacts with MHC-I in both early and late endosomes, and at the trans-Golgi network, but that the Nef:MHC-I interaction is not detectable in lysosomes. Interestingly, we show that Nef depletes the amount of MHC-I in Rab11 positive recycling endosomes and that a functional early endosomal compartment is required for Nef-dependent MHC-I downregulation. This Nef-mediated rerouting eventually sequesters MHC-I at the trans-Golgi network. Taken together, these results, demonstrate for the first time, the specific endocytic compartments utilized by Nef to orchestrate MHC-I downregulation and support a model that results in the sequestration of MHC-I by Nef in a non-degradative compartment.

## Results

### Bimolecular fluorescence complementation microscopy detects a Nef:MHC-I complex in cells

**Figure 1:**
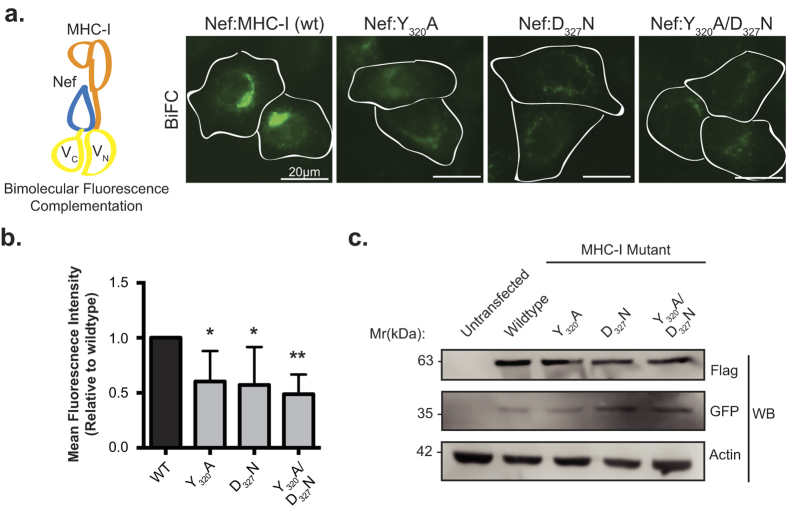
Bimolecular fluorescence complementation is observed between Nef and MHC-I. (**a**) Left: Schematic representation of the BiFC reporter system. Right: Nef-V_C_ and either wildtype MHC-I-V_N_-Flag or the indicated mutants were transfected into HeLa cells, 24 hrs later cells were fixed, and BiFC fluorescence (green) was observed under the FITC channel. Scale bars represent 20 μm. (**b**) Fluorescence intensities of Nef and MHC-I-V_N_-Flag positive cells were quantified in ImageJ, minus the background signal, to observe a decrease in fluorescence in the presence of the MHC-I mutations (n = 100, *indicates p-value < 0.05, **indicates p-value < 0.01). (**c**) Flag and GFP specific Western blots were conducted to ensure equal expression of both the MHC-I-V_N_-Flag mutants and Nef-V_C_, respectively. An actin specific Western blot was conducted as a loading control.

The immunoevasive capabilities of HIV-1 are largely mediated by the ability of Nef to remove MHC-I from the cell surface^24^. The crystal structure of a Nef:MHC-I complex revealed that this interaction is stabilized by AP-1, demonstrating that Nef, MHC-I and AP-1 are able to form a ternary complex^24^. To validate the Nef:MHC-I interaction in cells, we performed a bimolecular fluorescence complementation (BiFC) assay. BiFC entails the expression of a split Venus fluorophore from two distinct plasmids in the form of fusion proteins, and results in a reconstituted, functional Venus fluorophore when the two fusion proteins are within 100 nm^20^. Co-transfection of HeLa cells with plasmids encoding Nef-V_C_ and MHC-I-V_N_-Flag, more specifically Nef and MHC-I fused to carboxy (V_C_) or amino (V_N_) fragments of Venus, respectively, revealed that Nef and MHC-I form a complex (Fig. 1a). In order to test for the requirement of AP-1 in the formation of the Nef:MHC-I complex in cells, we tested Nef:MHC-I BiFC with MHC-I encoding mutations in residues previously implicated in interacting with AP-1 when MHC-I is in complex with Nef (MHC-I Y_320_ or D_327_). Co-transfection of plasmids encoding MHC-I Y_320_A-V_N_ or MHC-I D_327_N-V_N_ and Nef-V_C_ into HeLa cells revealed a decrease in the BiFC signal relative to wild type MHC-I (Fig. 1a), as measured by Venus mean fluorescence intensity in cells expressing both Nef and Flag tagged MHC-I (Fig. 1b). Similarly, co-expression of Nef-V_C_ and a MHC-I double mutant (MHC-I Y_320_A/D_327_N) resulted in a 2-fold reduction in BiFC signal (Fig. 1a,b), indicating that interactions between MHC-I and AP-1 are critical to observe a Nef:MHC-I BiFC signal, supporting the formation of a Nef:MHC-I:AP-1 ternary complex in cells. Importantly, the reductions in BiFC signal with the various MHC-I mutants were not due to differences in protein expression, as revealed by Western blot analysis (Fig. 1c). Furthermore, we demonstrate that the fusion of V_N_ or V_C_ to MHC-I or Nef respectively, did not alter protein function with respect to downregulation, as we previously demonstrated^22^. Specifically, wildtype MHC-I-V_N_ is downregulated by Nef (Supplemental Fig. S1), unlike the double mutant (Y_320_A/D_327_N) (Supplemental Fig. S1). Similarly, Nef-V_C_ is able to efficiently downregulate cell surface MHC-I (Supplemental Fig. S1).

### Nef targets recycling MHC-I prior to transit through a Rab11 compartment

**Figure 2:**
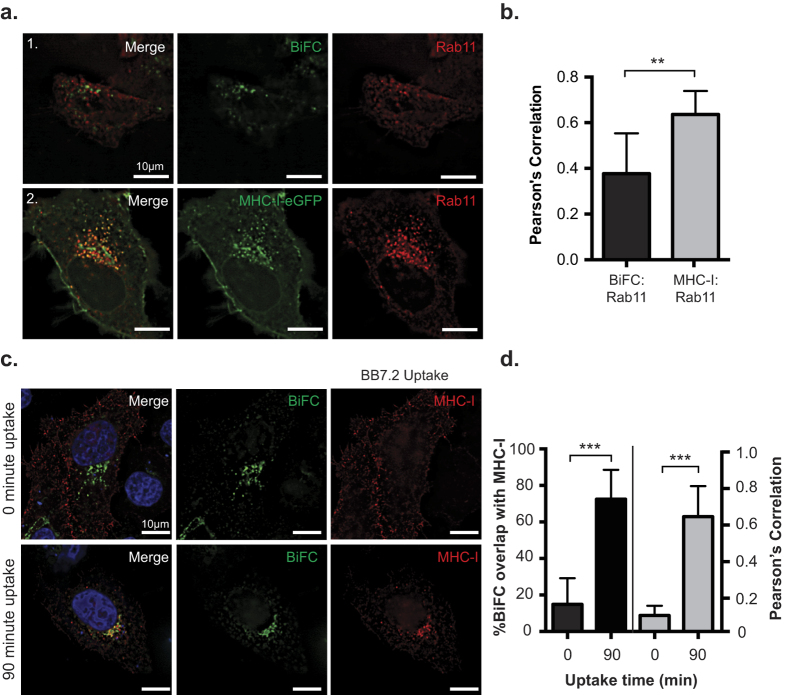
Nef prevents MHC-I from entering into a Rab11 dependent recycling route. (**a**) MHC-I-eGFP (panel 1) or Nef-V_C_ and MHC-I-V_N_-Flag (panel 2) and dsRed-Rab11 were co-transfected into HeLa cells. 24 hrs post transfection, cells were fixed and mounted onto coverslips. GFP and BiFC fluorescence was observed under the FITC channel, and dsRed-Rab11a was observed under the Cy3 channel. (**b**) Co-localization was quantified by using the Pearson’s correlation through the JaCoP Plug-in on ImageJ. (**c**) MHC-I (BB7.2) uptake experiments were performed as described in the materials and methods. BiFC signal is visualized in green, and MHC-I uptake was pseudocolored in red, nuclei were counterstained in blue. (**d**) Percent of co-localization (left axis and black bars), and Pearson’s correlation (right axis and grey bars) were determined using the Mander’s and Pearson’s correlation respectively through the JaCoP Plug-in on ImageJ. Error bars were calculated by quantification of at least 50 cells between 3 independent experiments. (**Indicates p-value < 0.01, ***indicates p-value < 0.001).

An obligate step in cellular homeostasis involves the rapid recycling of MHC-I to and from the cell surface in Rab11-positive recycling endosomes^25^. We first tested if Nef disrupts this rapid recycling step. To test this, we co-transfected HeLa cells with plasmids encoding MHC-I-eGFP and Rab11a-dsRed (Fig. 2a; panel 2), which labels recycling endosomes^26^. We then compared this to cells expressing Nef:MHC-I BiFC and Rab11-dsRed, in order to focus specifically on the MHC-I molecules that are targeted by Nef (Fig. 2a: panel 1). Pearson’s correlation analysis revealed that there was ~1.6 fold less co-localization between Rab11-dsRed and Nef:MHC-I BiFC than between Rab11-dsRed and MHC-I-eGFP, suggesting that Nef targets MHC-I prior to MHC-I entering a Rab11 dependent recycling route. Furthermore, an antibody uptake experiment confirmed that the majority of MHC-I that is being targeted by Nef originates from the cell surface (Fig. 2c), as the Nef-MHC-I BiFC signal originating from Nef-V_C_ and MHC-I-V_N_-Flag strongly co-localized with the BB7.2 antibody (Fig. 2d).

### Nef interacts with MHC-I within an early endosomal compartment

**Figure 3:**
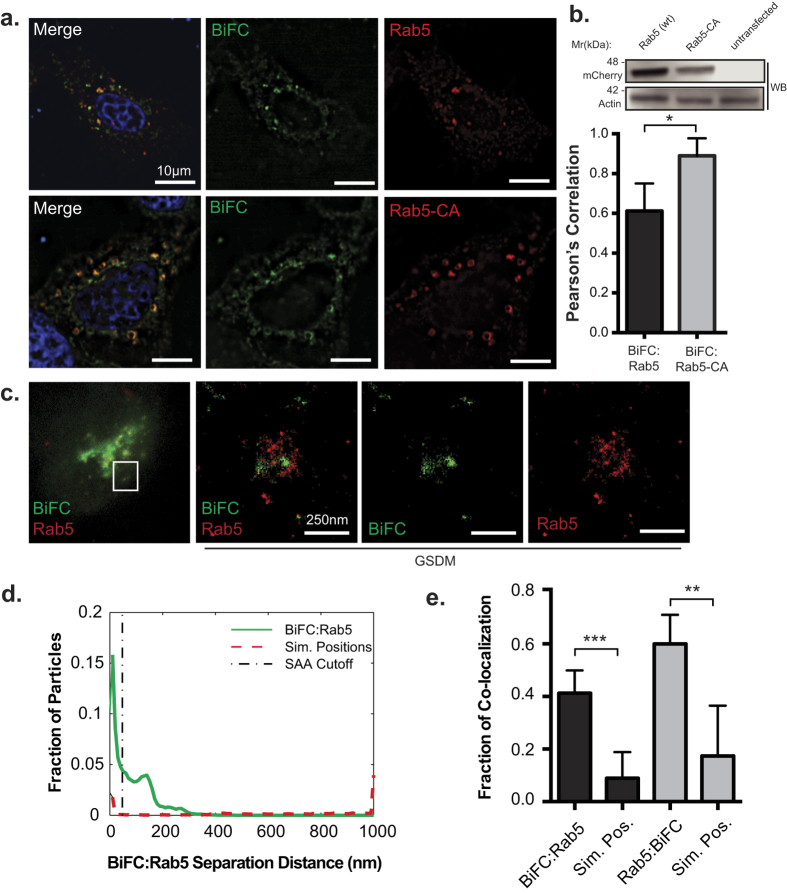
Nef:MHC-I interaction occurs within Rab5-positive early endosomes. (**a**) HeLa cells were co-transfected with plasmids encoding Nef-V_C_, MHC-I-V_N_-Flag and the indicated mCherry-tagged Rab5 constructs (wildtype and constitutively active (CA)). Twenty-four hours post transfection cells were fixed and mounted with DAPI Fluoromount-G. BiFC fluorescence (green) was detected under the FITC channel, while the mCherry-tagged Rab5 constructs were visualized under the Cy3 filter settings. Nuclei were visualized under the DAPI channel (scale bars represent 10 μm). (**b**) Co-localization of Nef:MHC-I BiFC with the mCherry tagged Rab5 constructs was quantified by the Pearson’s correlation through the JaCoP Plug-in on ImageJ. Error bars were calculated by quantification of at least 25 cells between 3 independent experiments (*Indicates p-value < 0.05). Western blot analysis for mCherry to confirm expression levels of Rab5 and Rab5-CA, with actin as a loading control (**c**) Cells were transfected with Nef-V_C_ and MHC-I-V_N_-Flag and immunostained for Rab5 and subsequently imaged utilizing ground state depletion microscopy (GSDM). (**d**) A histogram plotting the intermolecular distances between the nearest neighbor, representing either BiFC:Rab5 (Solid line) or BiFC:Simulated random positions (Sim. Positions; Dashed line). (**e**) A graphical representation of the fraction of co-localized particles observed in (**d**) which were observed to be under the cut-off value of ~40 nm. Error bars were calculated by quantification of 10 cells in 3 independent experiments (**indicates p-value < 0.01, ***indicates p-value < 0.001).

Since Nef reroutes cell surface MHC-I away from recycling endosomes (Fig. 2a,b), we next tested if a Nef:MHC-I complex would be located in early endosomes. Early endosomes constitute the initial compartment implicated in the endocytosis and sorting of cell surface cargo^15^,^27^. We co-transfected HeLa cells with plasmids encoding Nef-V_C_, MHC-I-V_N_-Flag and the early endosomal effector molecule mCherry-Rab5. Fluorescence microscopy analysis revealed that the Nef:MHC-I interaction co-localized with mCherry-Rab5, suggesting that the complex is present in early endosomes (Fig. 3a; Pearson’s = 0.612). Furthermore, in order to confirm that the Nef:MHC-I complex localizes to early endosomes, we co-expressed a constitutively active mCherry-tagged Rab5 molecule (mCherry-Rab5-CA; mCherry-Rab5 Q67L) with Nef-V_C_ and MHC-I-V_N_-Flag. Expression of Rab5-CA has previously been associated with a build-up of cargo molecules in early endosomes resulting in their subsequent enlargement^28^. Rab5-CA expression modified the co-localization of the Nef:MHC-I BiFC signal, wherein the BiFC signal was more prominent within Rab5 positive early endosomes (Pearson’s = 0.89), confirming that these vesicles are indeed a transiting point for Nef:MHC-I complexes (Fig. 3a,b). Furthermore, expression of Rab5-CA reduced Nef’s ability to downregulate MHC-I, demonstrating that a functional early endosomal compartment through which Nef-MHC-I complexes can transit is required for optimal downregulation of MHC-I from the cell surface (Supplemental Fig. S2).

Current resolution limits of conventional widefield microscopy do not allow for the visualization of discrete vesicular structures. To determine whether the Nef:MHC-I complex is contained within Rab5 early endosomes, we imaged the Rab5-dependent sorting step using the super-resolution microscopy technique of ground-state depletion microscopy (GSDM). This technique allows for visualization of cellular compartments with a resolution 10X greater than conventional microscopy^29^. To visualize early endosomes by GSDM, HeLa cells were transfected with the BiFC plasmids (Nef-V_C_ and MHC-I-V_N_-Flag), and immunostained for Rab5. The 10–25 fold gain in resolution provided by GSDM allowed for the detection of single Nef:MHC-I positive vesicles coated with the early endosomal effector Rab5 (Fig. 3c). Quantification of the association between Nef:MHC-I and Rab5 using the spatial association algorithm (SAA), which calculates the distance between punctate structures of different acquisition channels to determine if they are interacting/co-localizing together^30^. The SAA analysis revealed that the association of the Nef:MHC-I BiFC complex with Rab5 is significantly greater than that of randomly simulated positions in either acquisition channel (Fig. 3d,e). Taken together, multiple imaging techniques have demonstrated the close localization of the Nef:MHC-I complex with early endosomes suggesting that this organelle plays a key role in the immunoevasive capabilities of HIV-1.

### Nef traffics MHC-I to a late endosomal compartment

**Figure 4:**
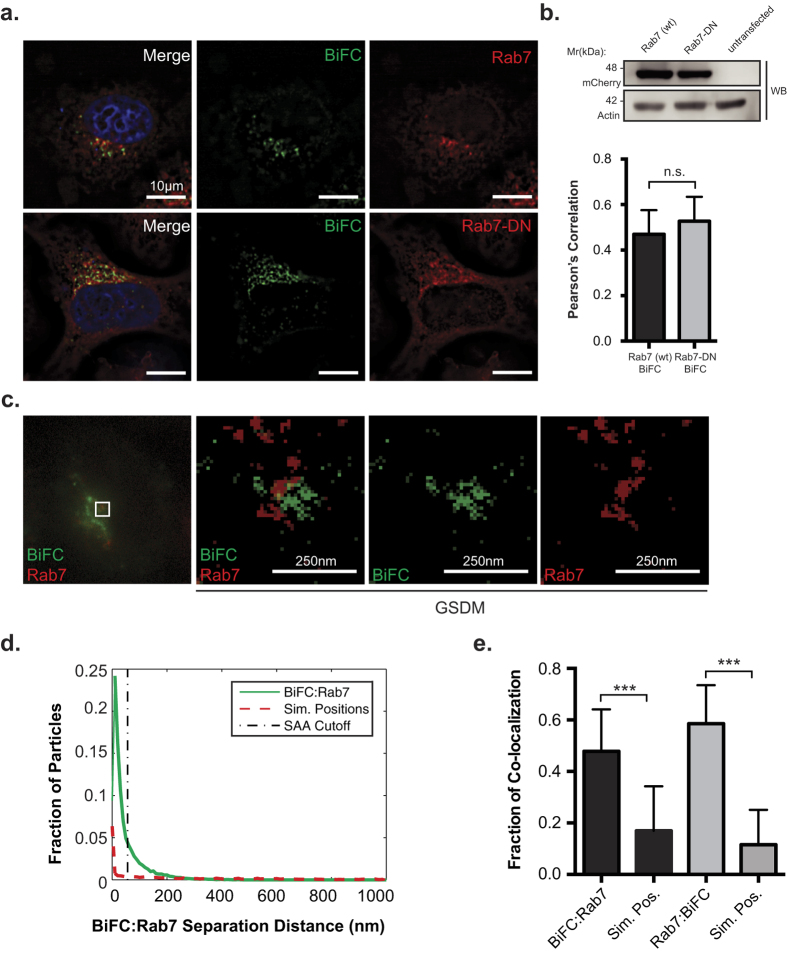
Nef:MHC-I interaction occurs within Rab7-positive late endosomes. (**a**) HeLa cells were co-transfected with Nef-V_C_ and MHC-I-V_N_-Flag encoding constructs along with constructs encoding wildtype mCherry-Rab7 or dominant negative mCherry-Rab7 (mCherry-Rab7-DN). BiFC fluorescence (green) was visualized under the FITC channel, mCherry-Rab7 (red) was detected under the Cy3 channel. Nuclei were stained with DAPI (scale bars represent 10 μm). (**b**) Co-localization was quantified by using the Pearson’s correlation through the JaCoP Plug-in on ImageJ. Error bars were calculated by quantification of at least 40 cells between 3 independent experiments. Western blot analysis for mCherry to confirm expression levels of Rab7 and Rab7-DN, with actin as a loading control. (**c**) Cells were transfected with Nef-V_C_ and MHC-I-V_N_-Flag and immunostained for Rab7 and subsequently imaged utilizing ground state depletion microscopy (GSDM). (**d**) A histogram plotting the intermolecular distances between the nearest neighbor, representing either BiFC:Rab7 (Solid line) or BiFC:Simulated random positions (Sim. Positions; Dashed line). (**e**) A graphical representation of the fraction of co-localized particles observed in (**d**) which were observed to be under the cut-off value of ~40 nm. Error bars were calculated by quantification at least 10 cells in 3 independent experiments (**indicates p-value < 0.01, ***indicates p-value < 0.001).

Early endosomes mature to form late endosomes within the endocytic network^14^. In order to test if the Nef:MHC-I complex is present in late endosomes we visualized the Nef:MHC-I BiFC signal in the presence of the late endosomal marker Rab7, an effector molecule specifically loaded onto late endosomes^31^. HeLa cells expressing Nef-V_C_ and MHC-I-V_N_-Flag were co-transfected with a plasmid encoding mCherry tagged wild type Rab7 (mCherry-Rab7). We observed that the Nef:MHC-I interaction occurs in late endosomes that are positive for mCherry-Rab7 (Fig. 4a). To further control for the Rab7 localization of Nef and MHC-I we utilized dominant-negative mCherry-tagged Rab7 (mCherry-Rab7-DN; Rab7 T22N). Previous expression of Rab7-DN has resulted in defects in trafficking of cargo transiting through late endosomes^32^. When testing the co-localization of the Nef:MHC-I BiFC with overexpressed mCherry-Rab7-DN, we observed no significant difference in colocalization between the wildtype and dominant-negative Rab7 (Fig. 4a,b). To confirm the late endosomal localization of the Nef:MHC-I complex, we utilized ground-state depletion super-resolution microscopy to gain the resolution needed to identify Rab7-positive vesicles containing the Nef:MHC-I complex (Fig. 4c). We observed that the Nef:MHC-I complex associated closely with Rab7. The SAA analysis revealed that the association of the Nef:MHC-I BiFC complex with Rab7 is significantly greater than that of randomly simulated positions in either acquisition channel (Fig. 4d,e). Taken together, these data demonstrate the localization of the Nef:MHC-I complex within Rab7 positive late endosomes, thereby implicating the late endosome in the re-routing of MHC-I away from the plasma membrane.

### Nef and MHC-I are not trafficked to the lysosome, but traffic to the trans-Golgi network

**Figure 5:**
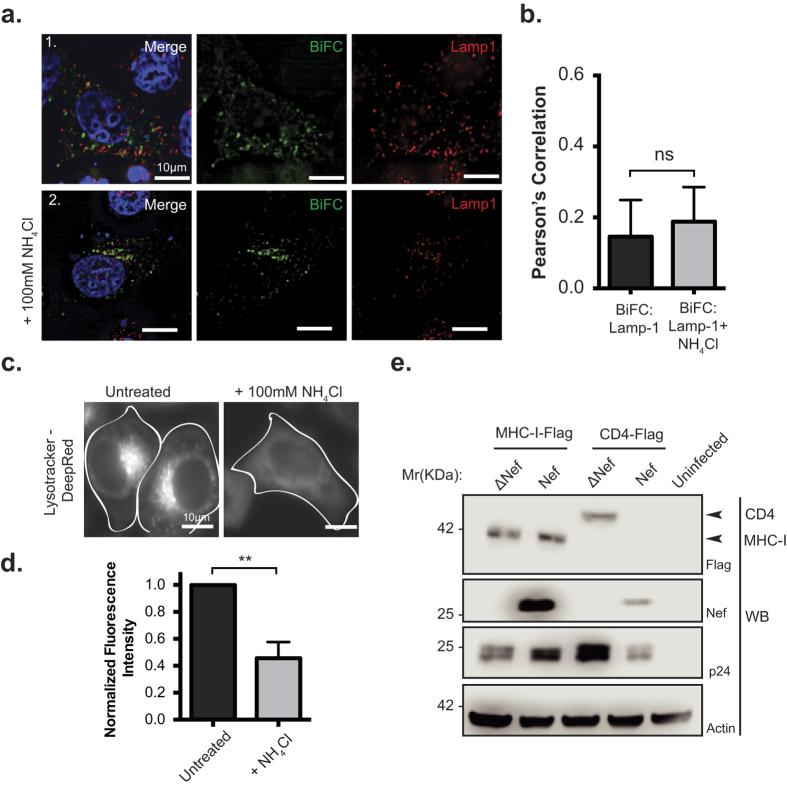
Nef:MHC-I interaction does not occur in lysosomes. (**a**) HeLa cells were co-transfected with Nef-V_C_ and MHC-I-V_N_-Flag constructs and immunostained for LAMP-1 (Panel 1), or, treated with 100 mM ammonium chloride (NH_4_Cl) for 3 hours prior to fixation and staining (Panel 2). BiFC fluorescence (green) was visualized under the FITC channel, whereas LAMP-1 stain was visualized under the Cy5 filter settings, and pseudo-colored red. Nuclei were stained with DAPI, and scale bars represent 10 μm. (**b**) Co-localization was quantified by the Pearson’s Correlation through the JaCoP Plug-in on ImageJ. Error bars were calculated by quantification of at least 25 cells between 3 independent experiments. (**c,d**) HeLa cells were treated with PBS, or 100 mM of ammonium chloride for 3 hours, and then treated with 10 μM Lysotracker Deep Red for 5 minutes. Live cells were then imaged at 37 °C in 5% CO_2_ and quantified for Lystotracker fluorescence. Error bars were calculated by quantification of at least 25 cells between 3 independent experiments. (**Indicates p-value < 0.01). (**e**) Sup-T1 cells were infected with F2A MHC-I-Flag-Nef/ΔNef or F2A-CD4-Flag-ΔVpu-Nef/ΔNef viruses. 48 hours post infection, cells were lysed and analyzed by Western blot. Anti-Flag detected total MHC-I-Flag and CD4-Flag, whereas anti-Nef antibody marked the presence or absence of Nef. Anti-p24 and anti-actin antibodies were used as infection and loading controls, respectively. A representative Western blot from 3 independent experiments is shown.

Consistent with the maturation of certain late endosomes to lysosomes^27^, we next tested if the Nef:MHC-I complex is present in lysosomes. HeLa cells overexpressing Nef-V_C_ and MHC-I-V_N_-Flag were immunostained with an antibody recognizing LAMP-1, a marker of lysosomes^33^ (Fig. 5a; panel 1). Immunofluorescence analysis revealed no significant co-localization of the Nef-MHC-I complex with lysosomes (Pearson’s = 0.15), suggesting that the complex does not traffic through this compartment. As lysosomes are a prime site of proteolytic degradation^34^,^35^, we sought to control for any degradation of Nef:MHC-I complexes that may occur within lysosomes and thereby mask our ability to detect these complexes within this compartment prior to imaging. Accordingly, HeLa cells were treated with ammonium chloride (NH_4_Cl) at 20 hours post transfection for 4 hours, which will block lysosomal degradation by increasing the cellular pH and thereby inactivating protease activity within lysosomes (Fig. 5a; panel 2)^35^. Despite increasing the cellular pH, we were unable to visualize the presence of the Nef:MHC-I complex within LAMP-1 positive lysosomes. In order to confirm that our NH_4_Cl treatment was sufficient to block lysosomal acidification we treated cells with Lysotracker, an agent retained in acidic compartments or we treated cells with complete media. Fluorescence intensity analysis revealed that Lysotracker dye fluorescence intensity was decreased, and not retained within distinct vesicular structures in the presence of NH_4_Cl, confirming that our NH_4_Cl treatment sufficiently inhibits the acidification of lysosomes (Fig. 5c,d).

In order to test the functional consequence of the Nef:MHC-I complex trafficking through early and late endosomes, we designed a lentiviral vector that expressed Flag-tagged MHC-I simultaneously with HIV-1 viral proteins (Fig. 5e). This vector system, which we have previously described^22^, utilizes the self-cleaving property of the 2 A peptide (F2A) from the foot and mouth virus to express MHC-I that is not linked to HIV-1 Gag/Pol. The infection of SupT1 cells with pNL4–3 F2A-MHC-I-Flag Nef and subsequent Western blotting demonstrated that the presence of Nef does not alter the protein expression levels of MHC-I, confirming that Nef does not direct MHC-I to degradative lysosomal compartment, consistent with previous experiments (Fig. 5e)^7^,^22^. Conversely, infection of SupT1 cells with a virus that overexpresses the CD4 receptor, pNL4–3 F2A-CD4-Flag ΔVpu Nef demonstrated that Nef is capable of reducing total cellular CD4 (Fig. 5e), which is consistent with previous reports of Nef-mediated degradation of CD4^36^. Thus, the functional consequence of Nef on MHC-I and CD4 expression is different. In addition, to confirm that Nef expression was not sufficient to traffic MHC-I to lysosomes, we expressed MHC-I-eGFP and Nef-mCherry under non-BiFC conditions and found no significant localization of MHC-I-eGFP with the LAMP-1 lysosomal marker in the presence of Nef, with or without ammonium chloride treatment (Supplemental Fig. S3).

**Figure 6:**
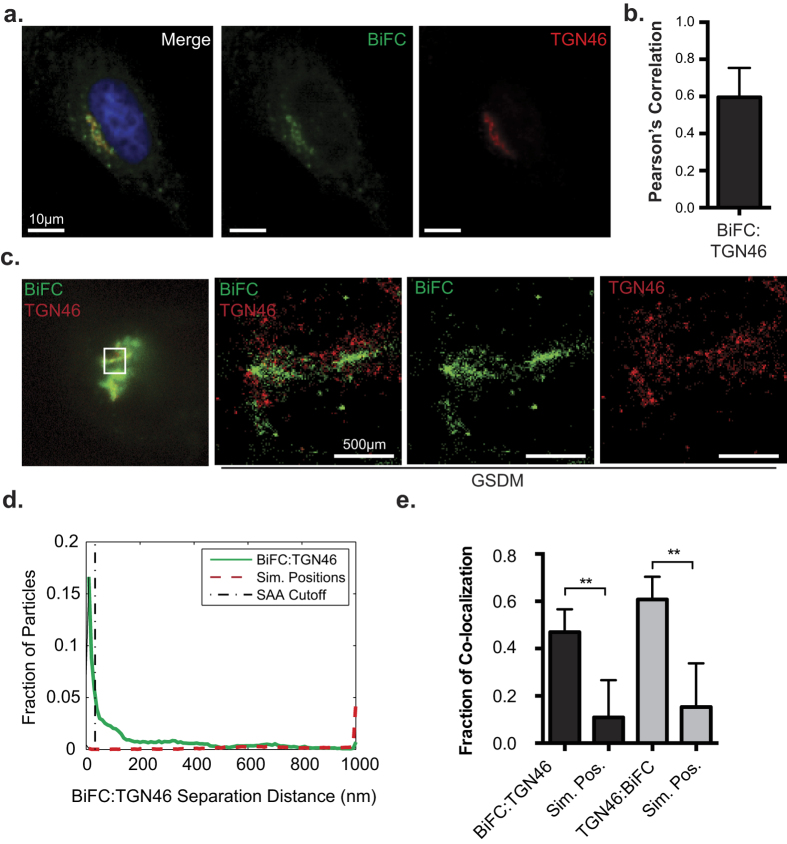
Nef targets MHC-I for sequestration within the trans-Golgi network. (**a**) Nef-V_C_ and MHC-I-V_N_-Flag were transfected into HeLa cells, and 24 hours later fixed and immunostained for TGN46. BiFC fluorescence (green) was observed under the FITC channel, and TGN46 (red) was observed under the Far-Red filters. (**b**) Co-localization was quantified by using the Pearson’s correlation through the JaCoP Plug-in on ImageJ. Error bars were calculated by quantification of at least 25 cells between 3 independent experiments. (*Indicates p-value < 0.05). (**c**) Cells were prepared as in (**a**) and imaged utilizing GSDM; scale bars represent 500 nm. (**d**) A histogram plotting the distance between the nearest neighbor, representing BiFC:TGN46 (Solid line) or BiFC:Simulated random positions (Sim. Positions; Dashed line). (**e**) A graphical representation of the fraction of co-localized particles observed in (**d**) which were observed to be under the cut-off value of ~40 nm. Error bars were calculated by quantification of 8 cells in 2 independent experiments (**indicates p-value < 0.01, ***indicates p-value < 0.001).

Since the Nef-MHC-I complex did not traffic to lysosomes we sought to identify an alternative subcellular localization for the complex. Previous studies have demonstrated that MHC-I is re-routed by Nef to a paranuclear compartment^37^. In order to visualize this compartment relative to the Nef:MHC-I complex, we counterstained HeLa cells co-expressing Nef-V_C_ and MHC-I-V_N_-Flag with a marker of the TGN, TGN46^38^ (Fig. 6a). A high degree of co-localization was observed between the Nef:MHC-I complex and TGN46 (Fig. 6b; Pearson’s = 0.60) suggesting that the Nef:MHC-I complex traffics to the TGN^38^. However, our previous work demonstrated that Nef localizes to an uncharacterized Golgi-proximal compartment that cannot be resolved from the TGN in conventional images^30^,^38^. As such, GSDM imaging was performed to map the localization of the Nef:MHC-I complexes with high precision. HeLa cells transfected with Nef-V_C_ and MHC-I-V_N_ and immunostained for the TGN marker TGN46 demonstrated the close association of the Nef:MHC-I complex with TGN46 in both the epifluorescence image, and the super-resolution image (Fig. 6c). Quantification of this complex demonstrated a significant increase in the association between the BiFC signal of the Nef:MHC-I complex with TGN46 compared to randomized images, confirming that Nef:MHC-I complexes traffic to the TGN (Fig. 6d,e).

## Discussion

In the present study, we have tracked a complex between the HIV-1 protein Nef and MHC-I within the endosomal network. Our results demonstrate that a Nef:MHC-I complex traffics to both early and late endosomes in addition to the paranuclear TGN compartment. Furthermore, Nef impedes MHC-I from recycling to the cell surface by re-routing MHC-I through both early endosomes and late endosomes and subsequently to the trans-Golgi network. Overall, this mechanism inhibits MHC-I molecules from presenting antigens extracellularly, contributing to the ability of HIV-1 to evade host immune surveillance^12^.

Nef has the capability to disrupt the normal membrane trafficking events that occur within cells. Indeed, Nef downregulates the cell surface expression of over 36 surface receptors^39^. Interestingly, there is some degree of specificity within this seemingly non-discriminatory razing of the cell surface topography. This specificity lies within the host membrane adaptor molecules that are used by Nef to internalize specific receptors. For example, the membrane adaptor molecule AP-1 interacts with MHC-I and is implicated in the Nef-dependent internalization of MHC-I^8^. Conversely, the related membrane adaptor molecule, AP-2, is critical for the Nef-dependent internalization of CD4^40^. Overall, these adaptor molecules are indispensible for the ability of Nef to bind and downregulate MHC-I and CD4 receptors. Structural studies have revealed that the cytoplasmic tails of MHC-I and CD4 mediate this specificity with adaptor molecules. Specifically, AP-1 recognizes the Yxxθ motif (where θ represents a bulky hydrophobic residue) in the MHC-I cytoplasmic tail, as demonstrated by the complex crystal structure between Nef:MHC-I and the mu-1 subunit of AP-1^10^. In contrast, structural modeling of the CD4 cytoplasmic tail has implicated the canonical di-leucine motif of CD4 to be necessary for its downregulation by Nef^41^. In this study, residues Y_320_ and D_327_ in the MHC-I cytoplasmic tail were deemed critical to maintain the Nef:MHC-I complex in cells (Fig. 1). As these residues were implicated in interacting with AP-1 within crystal structures, this supports the formation of a Nef:MHC-1:AP-1 ternary complex within cells, in agreement with previous studies utilizing co-immunoprecipiation^8^,^42^. Functionally, the differences in Nef dependent internalization of CD4 and MHC-I are linked to the distinction that AP-2 dependent trafficking is linked to degradative compartments whereas AP-1 dependent trafficking is not^7^,^40^,^43^. We confirmed this assertion as Nef and MHC-I complexes were not directed to degradative compartments (Fig. 5a) and MHC-I was not degraded (Fig. 5e), whereas Nef mediated the degradation of CD4 as previously described (Fig. 5e and ref. 40).

BiFC is a powerful technique that demonstrates the interaction or close proximity of two proteins within a cell. Previously, BiFC was used to illustrate the interaction between Nef and the membrane trafficking regulator PACS proteins, PACS-1 and PACS-2, in late and early endosomes, respectively^2^. Thus, the BiFC interaction between Nef and MHC-I provides a platform to study the various models of Nef-dependent MHC-I downregulation. Our results demonstrate that newly endocytosed MHC-I originating from the cell surface is targeted by Nef (Fig. 2c,d). Further experiments highlight the presence of the Nef:MHC-I complex throughout the early and late endosomes, and demonstrate the importance of the early endosomal effector molecule Rab5 in this process (Fig. 3 and Supplemental Fig. S2). As Rab5 functions to sort cargo early upon endocytosis^44^, it is logical that the expression of Rab5-CA, which disrupts the maturation of endosomes^44^, disrupts Nef-dependent MHC-I cell surface downregulation^3^. A possible mechanism governing this trafficking step may rely on a ternary complex between Nef, MHC-I and the PACS proteins, as previously demonstrated biochemically for PACS-1^2^. Interestingly, our results are directly linked to the signaling model of Nef-dependent MHC-I downregulation^7^, which is dependent on the PACS protein dependent endocytosis of MHC-I from the cell surface and not a block of MHC-I trafficking from the endoplasmic reticulum to plasma membrane which is linked to the Nef-dependent degradation of MHC-I in lysosomes^45^. We failed to observe the localization of the Nef:MHC-I complex in lysosomes even upon rendering these compartments more basic (Fig. 5a–c), suggesting that our experiments closely mimicked early time points in an infection^7^. This trafficking route would not result in the degradation of MHC-I, a phenomenon observed readily in other viral infections, such as Kaposi-sarcoma related herpesvirus infections, which mediates degradation of MHC-I by the K3 and K5 proteins^46^. Instead, the Nef:MHC-I complex was routed from Rab5 positive early endosomes to Rab7 positive late endosomes and the TGN and these interactions were observed at the 20 nm resolution offered by GSDM, unequivocally defining the subcellular route undertaken by MHC-I in the presence of Nef (Figs 3, 4 and 6). Moreover, the localization of the Nef-MHC-I complex in Rab7 positive late endosomes is reminiscent of the Nef dependent-localization of SERINC5 within this compartment^47^,^48^. SERINC5 was recently identified as a host cellular antiretroviral factor that inhibits HIV-1 replication^48^. Thus, it appears that Rab7-positive late endosomes are used by Nef to block both infectivity and the CTL response. Overall, Rab7 late endosomes are compartments used by multiple viruses for various steps of the viral infectious cycle including entry^49^,^50^ and would also represent a central compartment used by Nef to enable key HIV-1 functions such as the sequestration of MHC-I in an intracellular compartment.

The Nef:MHC-I interaction was also observed in the trans-Golgi network, consistent with the Nef and AP-1 dependent sequestration of MHC-I in a paranuclear compartment (Fig. 6a)^37^. We postulate that this compartment contains the re-routed MHC-I that has been pulled away by Nef from the cell surface. Previous studies by Blagoveshchenskaya *et al*. demonstrated that Nef increased the rates of endocytosed MHC-I in an Arf6 dependent manner^3^. Herein, we demonstrate that this endocytosed MHC-I localizes to the TGN in complex with Nef (Fig. 6). Indeed, we show that MHC-I is excluded from Rab11-positive recycling endosomes when in complex with Nef, suggesting that this represents a membrane trafficking junction point that differs between cells that do or do not express Nef (Fig. 2a,b). In uninfected cells, normal MHC-I recycling occurs via Rab11-positive compartments to enable antigen cross presentation, while maintaining physiological MHC-I levels on the cell surface^51^,^52^.

Overall, we propose a model for the Nef-dependent downregulation of cell surface MHC-I that highlights the cellular compartments that are subverted by Nef in order to coordinate the removal of MHC-I away from the cell surface. Our results demonstrate that a Nef:MHC-I complex traffics to both early and late endosomes in addition to the paranuclear TGN compartment. Further studies will be aimed at determining whether the transition between early and late endosomes is required for MHC-I sequestration, or if sequestration of MHC-I is independent of endosomal maturation. This work builds on the model of Nef-mediated MHC-I downregulation by identifying key cellular compartments in which Nef targets MHC-I. Understanding how Nef targets MHC-I will lead to new insights into how viruses mediate immune evasion, and specifically how HIV-1 persists within the infected host.

## Methods

### Cells

HeLa and HEK-293T cells (ATCC, Manassas, VA) were grown in complete DMEM (HyClone, Logan, UT) containing 10% fetal bovine serum (FBS) (Wisent, Montreal, Canada) and 100 μg/ml penicillin-streptomycin (HyClone). Sup-T1 cells were grown in complete RPMI (HyClone) containing 10% FBS and 10 μM L-Glutamine (HyClone). All cell lines were grown at 37 °C in the presence of 5% CO_2_ and sub-cultured in accordance with supplier’s recommendations.

### Plasmids

HLA-A2 cDNA (provided by Dr. G. Thomas, University of Pittsburgh Medical School) was subcloned into a pcDNA 3.1 (+) plasmid encoding the N-terminal portion of the Venus fluorophore (V_N_ 1–173), as previously described^22^. NL4.3 *nef* was subcloned into a pV_C_-N1 backbone plasmid encoding the C-terminal portion of the split Venus fluorophore (V_C_ 155–273). HLA-A2 mutants were generated using overlap extension polymerase chain reaction. Expression vectors encoding mCherry-Rab5, mCherry-Rab5-DN, mCherry-Rab5-CA, mCherry-Rab7, mCherry-Rab7-DN, dsRed-Rab11a were provided by Dr. R. Flanagan, UWO and were previously described^28^,^53^. The MHC-I-eGFP plasmid was generated by subcloning the HLA-A2 gene into a peGFP-N1 (Clontech) using EcoRI and BamHI restriction digest enzymes. Viral vectors: pNL4.3 F2A-CD4-Flag Δvpu Nef/ΔNef and pNL4.3 F2A MHC-I-MHC-I-Flag Nef/ΔNef were generated by sub cloning MHC-I-Flag and CD4-Flag into the previously described base vector pNL4.3 F2A-X-Nef/ΔNef 22.

### Transfections

For BiFC and subcellular localization studies, 2.5×10^5^ HeLa cells were seeded onto coverslips and 24 hours later plasmids were transfected into cells at equal molar ratios using PolyJet transfection reagent (FroggaBio, Toronto, Canada). Twenty-four hours post transfection cells were incubated for one hour at room temperature to allow for fluorophore maturation, as described previously^22^. Subsequently, cells were fixed in 4% PFA and prepared for immunofluorescence as described below.

### Western Blots

For analysis of BiFC protein expression, HeLa cells were transfected with the specified BiFC vectors, incubated for 24 hours, washed once with phosphate buffered saline (PBS) and lysed in lysis buffer (0.5 M HEPES, 1.25 M NaCl, 1 M MgCl_2_, 0.25 M EDTA, 0.1% Triton X-100, 1X complete Protease inhibitor Tablets (Roche, Indianapolis, IN)). Cells were incubated on a rotator for 20 minutes at 4 °C before removing insoluble cellular debris by centrifugation at 20,000× g for 20 minutes. Lysates were boiled at 98 °C in 5X SDS-PAGE sample buffer (0.312 M Tris pH 6.8, 25% 2-Mercaptoethanol, 50% glycerol, 10% SDS) and proteins were separated on a 12% SDS-PAGE gel and subsequently transferred to nitrocellulose membranes. Membranes were blocked in 5% non-fat skimmed milk (BioShop Canada, Burlington, Canada) in TBST containing 0.1% Triton X-100 for 1 hour, then incubated overnight at 4 °C with various antibodies: rabbit anti-Nef polyclonal antibody (1:2000; catalog number 2949, NIH AIDS Research and Reference Reagent Program, USA^54^, rat anti-DYKDDDK monoclonal IgG (1:2500; BioLegend, San Diego, CA), anti-p24 (1:800; catalog number 4121, NIH AIDS Research and Reference Reagent Program, USA) and anti-Actin (1:2000; Thermo Scientific). Membranes were then washed and incubated for two hours with the appropriate species-specific HRP-conjugated antibodies (1:3000; Thermo Scientific). All blots were developed and quantified using ECL substrates (Millipore Inc., Billerica, MA) and a C-DiGit chemiluminescence Western blot scanner (LI-COR Biosciences, Lincoln, NE). To test Nef’s ability to mediate MHC-I or CD4 expression, we utilized our previously described viral vector system^22^ to express MHC-I-Flag or CD4-Flag, in the presence or absence of Nef. The resulting vectors: pNL4.3 F2A MHC-I-Flag Nef/ΔΝef or pNL4.3 F2A CD4-Flag Δvpu Nef/ΔNef were used to generate pseudoviral particles as previously described^22^. Sup-T1 cells were subsequently infected, and 48 hours post infection, cells were lysed and levels of MHC-I and CD4 were analyzed by Western blot as described above.

### Immunofluorescence

For subcellular localization studies, transfections were performed as described above. Cells used in BiFC studies were incubated at room temperature for 1 hour prior to fixation to allow the reconstituted fluorophore to mature^22^. All cells were fixed by washing twice with PBS, incubating for 20 minutes in 4% PFA and subsequently washing three times in PBS. To stain for the split fluorophore halves and various intracellular compartments, fixed HeLa cells were incubated in permeabilization/blocking buffer (5% BSA in PBS and 0.2% Triton X-100) for 1 hour. Cells were then incubated with the appropriate antibodies diluted in blocking buffer for 2 hours (anti-Rab5 (Cell Signaling); 1:200, anti-Rab7 (Santa-Cruz); 1:100, anti-LAMP-1 (DSHB); 1:200, anti-TGN46 (Sigma Aldrich); 1:200, anti-Flag (Biolegend): 1:400). Cells were subsequently washed three times in blocking buffer and incubated with the appropriate secondary antibody diluted in blocking buffer (donkey anti-rabbit AlexaFluor 647 or donkey anti-mouse AlexaFluor 647 (1:1000; Jackson ImmunoResearch)) for 2 hours at room temperature. Finally, cells were washed three times in PBS (3 minute each) and mounted onto glass slides using Fluormount-G or DAPI-fluoromount-G (Southern Biotech, Birmingham, AL). Fluorescence intensity of the BiFC signal was measured by selecting cells positive for both Nef and Flag-tagged MHC-I, then subtracting the background fluorescence using ImageJ^55^,^56^. To test the localization of MHC-I-eGFP with LAMP-1, cells were transfected with MHC-I-eGFP with Nef-mCherry or mCherry and immunostained as above. Co-localization of MHC-I-eGFP and LAMP-I was then measured in cells expressing either Nef-mCherry or mCherry in the presence or absence of ammonium chloride.

### Antibody Uptake

HeLa cells were transfected with equal molar amounts of MHC-I-V_N_-Flag and Nef-V_C_. Twenty-four hours post-transfection cells were washed with ice cold PBS, and subsequently anti-HLA-A2 (BB7.2; Biolegend) antibody was added at a dilution of 1:300. Antibody was allowed to bind for 20 minutes at 4 °C. Following antibody binding, cells were washed 3X with cold PBS, and either fixed (time 0 minutes), or supplemented with warm complete media and incubated for 90 minutes at 37 °C. Cells were then fixed, permeablized, and immunostained with donkey anti-mouse AlexaFluor 647 (1:400; Jackson ImmunoResearch) secondary antibody for 2 hours to detect the internalized antibody. Coverslips were then washed 3X in PBS and mounted using DAPI-Fluoromount-G (Southern Biotech).

### Ammonium chloride treatment of cells to assess lysosomal trafficking

For assessing lysosomal subcellular localization, staining was performed as described above with anti-human LAMP-1 1:200 (Developmental Studies Hybridoma Bank; Iowa City, IA). Ammonium chloride was added to a concentration of 100 mM to prevent lysosomal acidification for 4 hours. To ensure the ammonium chloride treatment affected lysosomal acidification, HeLa cells treated in an equivalent manner were stained with Lysotracker DeepRed (10 μM; Life Technologies). Live cells were then imaged, and mean fluorescence intensity was measured using ImageJ.

### Microscopy

Cells were viewed on a Leica DMI6000 B at 63X or 100X magnification using the FITC, Cy3, CY5 and DAPI filter settings and imaged with a Hamamatsu Photometrics Delta Evolve camera. Images were subsequently deconvolved using the Advanced Fluorescence Deconvolution (Lecia, Wetzlar, Germany) application on the Leica Application Suite software. Co-localization analysis was conducted using Pearson’s Correlation from the ImageJ plugin JACoP, as described previously^57^.

Super-resolution imaging was performed as previously described^30^. Briefly, HeLa cells were transfected with plasmid DNA, and 24 hours later immunostained as described above. Prior to imaging, cells were mounted in a depression slide containing 100 mM cysteamine (Sigma) buffer in PBS. Coverslips were sealed with Twinsil (Picodent), and imaged within 4 hours of mounting. All images were acquired using a Lecia SR GSD microscope with the 100X/1.43 NA objective lens containing an additional 1.6X magnifier. Fluorophores were excited using 125 mW–250 mW lasers (488, 532 and 647 nm) and a 30 mW backpumping 405 nm laser. Channels were acquired sequentially at 100 fps for 7,000–15,000 frames, with minor adjustments on laser power and backpumping to maintain 10 to 30 active fluorophores per frame. The resulting images were exported and molecule position files were converted to ASCII for analysis. Intermolecular interactions and localization within labeled endocytic compartments was quantified using spatial association analysis (SAA), performed using our custom-written MIiSR software^30^, with datasets filtered to remove molecules detected with a precision of <25 nm prior to analysis. All interactions were validated by comparing the observed degree of interaction to that observed in 10 images containing the same number of particles randomly scattered or an image of equal area.

### Flow cytometry

To test the functional significance of disrupting Rab5, HeLa cells were transfected with mCherry-tagged Rab5-CA or Rab5 (wt) constructs in conjunction with Nef-eGFP or empty eGFP encoding backbone. Twenty-four hours post transfection, cells were trypsinized, washed twice with PBS and fixed in 2% PFA for 15 minutes. Following fixation, cells were washed in FACS buffer (0.5% FBS and 50 mM EDTA in PBS) and stained with W6/32 anti-MHC-I (1:4000) antibody for 30 minutes (provided by D. Johnson, Oregon Health and Science University). Cells were then washed twice and stained with donkey anti-mouse AlexaFluor 647 (1:1000) for 20 minutes, followed by two more washes in FACS buffer. Flow cytometry was then performed using a BD FACSCanto (BD Biosciences) and the geometric mean fluorescence intensity of AlexaFluor 647 (MHC-I) was determined for mCherry and eGFP positive cells.

To test the functionality of the MHC-I-V_N_-Flag fusion proteins, we transfected HeLa cells with vectors encoding MHC-I-V_N_-Flag or MHC-I-Y_320_A/D_327_N-V_N_-Flag in combination with either Nef-eGFP or eGFP alone. Twenty-four hours post transfection cells, were stained with BB7.2 anti-HLA-A2 (1:4000) and donkey anti-mouse AlexaFluor 647 (1:2000), as above, to specifically detect cell surface MHC-I fusion proteins. Cells were then analyzed by flow cytometry as above and geometric mean fluorescence intensity of AlexaFluor 647 (MHC-I) was determined for eGFP and AlexaFluor 647 positive cells.

To test the functionality of Nef-V_C_, HeLa cells were transfected with vectors encoding Nef-V_C_ and eGFP as a transfection control, or a vector encoding eGFP alone. GFP positive cells were subsequently analyzed for cell surface MHC-I levels by flow cytometry using W6/32 anti-MHC-I antibody as described above.

## Additional Information

**How to cite this article**: Dirk, B. S. *et al*. HIV-1 Nef sequesters MHC-I intracellularly by targeting early stages of endocytosis and recycling. *Sci. Rep.* **6**, 37021; doi: 10.1038/srep37021 (2016).

**Publisher’s note:** Springer Nature remains neutral with regard to jurisdictional claims in published maps and institutional affiliations.

## Supplementary Material

Supplementary Information

## References

[b1] Malim M. H. & Emerman M. HIV-1 accessory proteins--ensuring viral survival in a hostile environment. Cell Host Microbe 3, 388–398, doi: S1931–3128(08)00126-1 (2008). 18541215 10.1016/j.chom.2008.04.008

[b2] Dikeakos J. D. . An interdomain binding site on HIV-1 Nef interacts with PACS-1 and PACS-2 on endosomes to down-regulate MHC-I. Molecular Biology of the Cell 23, 2184–2197, doi: mbc.E11-11-0928 (2012). 22496420 10.1091/mbc.E11-11-0928 PMC3364181

[b3] Blagoveshchenskaya A. D., Thomas L., Feliciangeli S. F., Hung C. H. & Thomas G. HIV-1 Nef downregulates MHC-I by a PACS-1- and PI3K-regulated ARF6 endocytic pathway. Cell 111, 853–866, doi: S0092867402011625 (2002). 12526811 10.1016/s0092-8674(02)01162-5

[b4] Collins D. R. & Collins K. L. HIV-1 accessory proteins adapt cellular adaptors to facilitate immune evasion. PLoS Pathog 10, e1003851 (2014). 24465204 10.1371/journal.ppat.1003851 PMC3900642

[b5] Collins K. L., Chen B. K., Kalams S. A., Walker B. D. & Baltimore D. HIV-1 Nef protein protects infected primary cells against killing by cytotoxic T lymphocytes. Nature 391, 397–401, doi: 10.1038/34929 (1998). 9450757

[b6] Pawlak E. N. & Dikeakos J. D. HIV-1 Nef: A Master Manipulator of the Membrane Trafficking Machinery Mediating Immune Evasion. Biochimica et Biophysica Acta (BBA)-General Subjects (2015). 10.1016/j.bbagen.2015.01.003 25585010

[b7] Dikeakos J. D. . Small molecule inhibition of HIV-1-induced MHC-I down-regulation identifies a temporally regulated switch in Nef action. Molecular Biology of the Cell 21, 3279–3292, doi: E10-05-0470 (2010). 20702582 10.1091/mbc.E10-05-0470 PMC2947465

[b8] Roeth J. F., Williams M., Kasper M. R., Filzen T. M. & Collins K. L. HIV-1 Nef disrupts MHC-I trafficking by recruiting AP-1 to the MHC-I cytoplasmic tail. J Cell Biol 167, 903–913, doi: jcb.200407031 (2004). 15569716 10.1083/jcb.200407031 PMC2172469

[b9] Wan L. . PACS-1 defines a novel gene family of cytosolic sorting proteins required for trans-Golgi network localization. Cell 94, 205–216 (1998). 9695949 10.1016/s0092-8674(00)81420-8

[b10] Jia X. . Structural basis of evasion of cellular adaptive immunity by HIV-1 Nef. Nat Struct Mol Biol 19, 701–706, doi: nsmb.2328 (2012). 22705789 10.1038/nsmb.2328 PMC3407041

[b11] Schaefer M. R., Wonderlich E. R., Roeth J. F., Leonard J. A. & Collins K. L. HIV-1 Nef targets MHC-I and CD4 for degradation via a final common beta-COP-dependent pathway in T cells. PLoS Pathog 4, e1000131 (2008). 18725938 10.1371/journal.ppat.1000131 PMC2515349

[b12] Tokarev A. & Guatelli J. Misdirection of membrane trafficking by HIV-1 Vpu and Nef: Keys to viral virulence and persistence. Cell Logist 1, 90–102, doi: 10.4161/cl.1.3.167082159-2780-1-3-4 (2011). 21922073 PMC3173656

[b13] Schwartz O., Maréchal V., Le Gall S., Lemonnier F. & Heard J.-M. Endocytosis of major histocompatibility complex class I molecules is induced by the HIV–1 Nef protein. Nature Medicine 2, 338–342 (1996). 10.1038/nm0396-338 8612235

[b14] Stoorvogel W., Strous G. J., Geuze H. J., Oorschot V. & Schwartzt A. L. Late endosomes derive from early endosomes by maturation. Cell 65, 417–427 (1991). 1850321 10.1016/0092-8674(91)90459-c

[b15] Rink J., Ghigo E., Kalaidzidis Y. & Zerial M. Rab conversion as a mechanism of progression from early to late endosomes. Cell 122, 735–749 (2005). 16143105 10.1016/j.cell.2005.06.043

[b16] Bucci C., Thomsen P., Nicoziani P., McCarthy J. & van Deurs B. Rab7: a key to lysosome biogenesis. Molecular Biology of the Cell 11, 467–480 (2000). 10679007 10.1091/mbc.11.2.467 PMC14786

[b17] Maxfield F. R. & McGraw T. E. Endocytic recycling. Nature Reviews Molecular Cell Biology 5, 121–132 (2004). 15040445 10.1038/nrm1315

[b18] Zerial M. & McBride H. Rab proteins as membrane organizers. Nature Reviews Molecular Cell Biology 2, 107–117 (2001). 11252952 10.1038/35052055

[b19] Kerppola T. K. Bimolecular fluorescence complementation (BiFC) analysis as a probe of protein interactions in living cells. Annu Rev Biophys 37, 465–487, doi: 10.1146/annurev.biophys.37.032807.125842 (2008). 18573091 PMC2829326

[b20] Kerppola T. K. Design and implementation of bimolecular fluorescence complementation (BiFC) assays for the visualization of protein interactions in living cells. Nature Protocols 1, 1278–1286 (2006). 17406412 10.1038/nprot.2006.201 PMC2518326

[b21] Dirk B. S., Heit B. & Dikeakos J. D. Visualizing Interactions Between HIV-1 Nef and Host Cellular Proteins Using Ground-State Depletion Microscopy. AIDS Research and Human Retroviruses 31, 671–672 (2015). 26061722 10.1089/aid.2014.0333 PMC4505775

[b22] Dirk B. S. . Viral Bimolecular Fluorescence Complementation: A Novel Tool to Study Intracellular Vesicular Trafficking Pathways. PloS One 10, e0125619 (2015). 25915798 10.1371/journal.pone.0125619 PMC4411132

[b23] Ye H., Choi H. J., Poe J. & Smithgall T. E. Oligomerization is required for HIV-1 Nef-induced activation of the Src family protein-tyrosine kinase, Hck. Biochemistry 43, 15775–15784, doi: 10.1021/bi048712f (2004). 15595833

[b24] Jia X. . Structural basis of evasion of cellular adaptive immunity by HIV-1 Nef. Nature Structural & Molecular Biology 19, 701–706 (2012). 10.1038/nsmb.2328 PMC3407041 22705789

[b25] Donaldson J. G. & Williams D. B. Intracellular assembly and trafficking of MHC class I molecules. Traffic 10, 1745–1752 (2009). 19761542 10.1111/j.1600-0854.2009.00979.x PMC2783374

[b26] Ullrich O., Reinsch S., Urbé S., Zerial M. & Parton R. G. Rab11 regulates recycling through the pericentriolar recycling endosome. The Journal of Cell Biology 135, 913–924 (1996). 8922376 10.1083/jcb.135.4.913 PMC2133374

[b27] Huotari J. & Helenius A. Endosome maturation. The EMBO Journal 30, 3481–3500 (2011). 21878991 10.1038/emboj.2011.286 PMC3181477

[b28] Stenmark H. . Inhibition of rab5 GTPase activity stimulates membrane fusion in endocytosis. The EMBO Journal 13, 1287 (1994). 8137813 10.1002/j.1460-2075.1994.tb06381.x PMC394944

[b29] Hell S. W. & Kroug M. Ground-state-depletion fluorscence microscopy: A concept for breaking the diffraction resolution limit. Applied Physics B 60, 495–497 (1995).

[b30] Caetano F. A. . MIiSR: Molecular Interactions in Super-Resolution Imaging Enables the Analysis of Protein Interactions, Dynamics and Formation of Multi-protein Structures. PLoS Comput Biol 11, e1004634 (2015). 26657340 10.1371/journal.pcbi.1004634 PMC4676698

[b31] Mukhopadhyay A., Funato K. & Stahl P. D. Rab7 regulates transport from early to late endocytic compartments in Xenopus oocytes. Journal of Biological Chemistry 272, 13055–13059 (1997). 9148916 10.1074/jbc.272.20.13055

[b32] Choudhury A. . Rab proteins mediate Golgi transport of caveola-internalized glycosphingolipids and correct lipid trafficking in Niemann-Pick C cells. The Journal of Clinical Investigation 109, 1541–1550 (2002). 12070301 10.1172/JCI15420 PMC151017

[b33] Rohrer J., Schweizer A., Russell D. & Kornfeld S. The targeting of Lamp1 to lysosomes is dependent on the spacing of its cytoplasmic tail tyrosine sorting motif relative to the membrane. The Journal of Cell Biology 132, 565–576 (1996). 8647888 10.1083/jcb.132.4.565 PMC2199866

[b34] Segal H. L. & Doyle D. J. Protein turnover and lysosome function. (Academic Press, 2014).

[b35] Cardelli J., Richardson J. & Miears D. Role of acidic intracellular compartments in the biosynthesis of Dictyostelium lysosomal enzymes. The weak bases ammonium chloride and chloroquine differentially affect proteolytic processing and sorting. Journal of Biological Chemistry 264, 3454–3463 (1989). 2492537

[b36] Piguet V. . Nef-induced CD4 degradation: a diacidic-based motif in Nef functions as a lysosomal targeting signal through the binding of β-COP in endosomes. Cell 97, 63–73 (1999). 10199403 10.1016/s0092-8674(00)80715-1

[b37] Piguet V. . HIV-1 Nef protein binds to the cellular protein PACS-1 to downregulate class I major histocompatibility complexes. Nature Cell Biology 2, 163–167 (2000). 10707087 10.1038/35004038 PMC1475706

[b38] Prescott A., Lucocq J. & Pannambalam V. TGN46 is localised in distinct domains of the HeLa cell Golgi apparatus. Eur. J. Cell Biol 72, 238–246 (1997). 9084986

[b39] Haller C. . HIV-1 Nef and Vpu are functionally redundant broad-spectrum modulators of cell surface receptors, including tetraspanins. Journal of Virology 88, 14241–14257 (2014). 25275127 10.1128/JVI.02333-14 PMC4249113

[b40] Chaudhuri R., Lindwasser O. W., Smith W. J., Hurley J. H. & Bonifacino J. S. Downregulation of CD4 by human immunodeficiency virus type 1 Nef is dependent on clathrin and involves direct interaction of Nef with the AP2 clathrin adaptor. J Virol 81, 3877–3890, doi: JVI.02725-06 (2007). 17267500 10.1128/JVI.02725-06 PMC1866153

[b41] Ren X., Park S. Y., Bonifacino J. S. & Hurley J. H. How HIV-1 Nef hijacks the AP-2 clathrin adaptor to downregulate CD4. Elife 3, e01754 (2014). 24473078 10.7554/eLife.01754 PMC3901399

[b42] Wonderlich E. R., Williams M. & Collins K. L. The tyrosine binding pocket in the adaptor protein 1 (AP-1) μ1 subunit is necessary for Nef to recruit AP-1 to the major histocompatibility complex class I cytoplasmic tail. Journal of Biological Chemistry 283, 3011–3022 (2008). 18073204 10.1074/jbc.M707760200

[b43] Janvier K. & Bonifacino J. S. Role of the endocytic machinery in the sorting of lysosome-associated membrane proteins. Molecular Biology of the Cell 16, 4231–4242, doi: 10.1091/mbc.E05-03-0213 (2005). 15987739 PMC1196333

[b44] Roberts R. . Endosome fusion in living cells overexpressing GFP-rab5. Journal of Cell Science 112, 3667–3675 (1999). 10523503 10.1242/jcs.112.21.3667

[b45] Kasper M. R. . HIV-1 Nef disrupts antigen presentation early in the secretory pathway. Journal of Biological Chemistry 280, 12840–12848 (2005). 15653685 10.1074/jbc.M413538200

[b46] Ishido S., Wang C., Lee B.-S., Cohen G. B. & Jung J. Downregulation of major histocompatibility complex class I molecules by Kaposi’s sarcoma-associated herpesvirus K3 and K5 proteins. Journal of Virology 74, 5300–5309 (2000). 10799607 10.1128/jvi.74.11.5300-5309.2000 PMC110885

[b47] Usami Y., Wu Y. & Göttlinger H. G. SERINC3 and SERINC5 restrict HIV-1 infectivity and are counteracted by Nef. Nature 526, 218–223 (2015). 26416733 10.1038/nature15400 PMC4600458

[b48] Rosa A. . HIV-1 Nef promotes infection by excluding SERINC5 from virion incorporation. Nature 526, 212–217 (2015). 26416734 10.1038/nature15399 PMC4861059

[b49] Saeed M. F., Kolokoltsov A. A., Albrecht T. & Davey R. A. Cellular entry of ebola virus involves uptake by a macropinocytosis-like mechanism and subsequent trafficking through early and late endosomes. PLoS Pathog 6, e1001110 (2010). 20862315 10.1371/journal.ppat.1001110 PMC2940741

[b50] Chu J. & Ng M. Infectious entry of West Nile virus occurs through a clathrin-mediated endocytic pathway. Journal of Virology 78, 10543–10555 (2004). 15367621 10.1128/JVI.78.19.10543-10555.2004 PMC516396

[b51] Nair-Gupta P. . TLR signals induce phagosomal MHC-I delivery from the endosomal recycling compartment to allow cross-presentation. Cell 158, 506–521 (2014). 25083866 10.1016/j.cell.2014.04.054 PMC4212008

[b52] Grommé M. . Recycling MHC class I molecules and endosomal peptide loading. Proceedings of the National Academy of Sciences 96, 10326–10331 (1999). 10.1073/pnas.96.18.10326 PMC17887 10468607

[b53] Rojas R. . Regulation of retromer recruitment to endosomes by sequential action of Rab5 and Rab7. The Journal of Cell Biology 183, 513–526 (2008). 18981234 10.1083/jcb.200804048 PMC2575791

[b54] Shugars D. C. . Analysis of human immunodeficiency virus type 1 nef gene sequences present *in vivo*. Journal of Virology 67, 4639–4650 (1993). 8043040 10.1128/jvi.67.8.4639-4650.1993 PMC237849

[b55] Schneider C. A., Rasband W. S. & Eliceiri K. W. NIH Image to ImageJ: 25 years of image analysis. Nat Methods 9, 671–675 (2012). 22930834 10.1038/nmeth.2089 PMC5554542

[b56] Abràmoff M. D., Magalhães P. J. & Ram S. J. Image processing with Image J. Biophotonics International 11, 36–42 (2004).

[b57] Bolte S. & Cordelieres F. A guided tour into subcellular colocalization analysis in light microscopy. Journal of Microscopy 224, 213–232 (2006). 17210054 10.1111/j.1365-2818.2006.01706.x

