# Supplementary material for: HIV-1 Nef sequesters MHC-I intracellularly by targeting early stages of endocytosis and recycling
Source: Sci Rep. 2016 Nov 14;6:37021. doi: 10.1038/srep37021 (PMC5107982; doi:10.1038/srep37021)
Supplement: Supplementary Information [file srep37021-s1.pdf]

HIV-1 Nef sequesters MHC-I intracellularly by targeting early stages of endocytosis and recycling

Brennan S. Dirk[1], Emily N. Pawlak[1], Aaron L. Johnson[1], Logan R. Van Nynatten[1], Rajesh A. Jacob[1], Bryan Heit[1] and Jimmy D. Dikeakos[1]

[1]Department of Microbiology and Immunology, The University of Western Ontario, Schulich School of Medicine and Dentistry. London, Ontario, Canada.

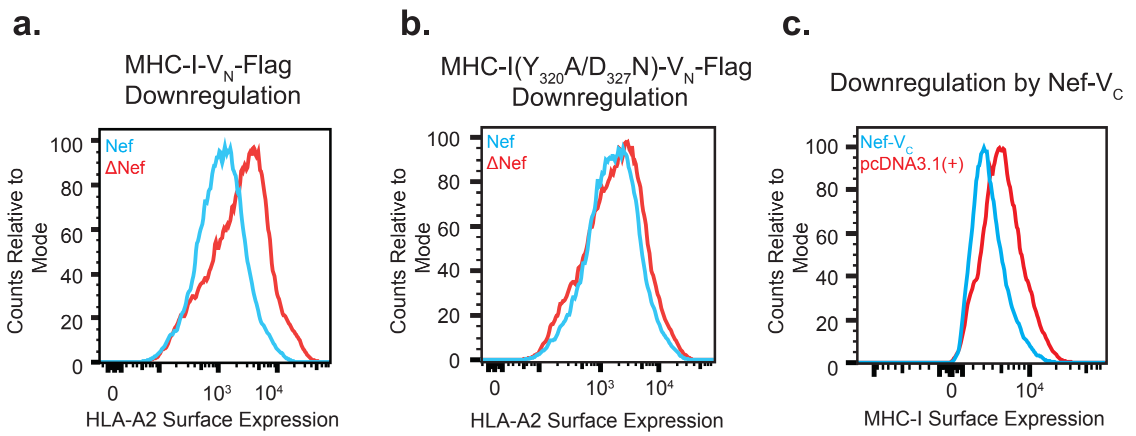

Supplemental Figure S1: Functionality of MHC-I and Nef fusion proteins.
(A and B) MHC-I-V_N-Flag or MHC-I-$Y_{320A}D_{327N}$-V_N-Flag and Nef-eGFP (blue line) or eGFP (red line) were co-transfected in HeLa cells and surface stained with BB7.2 (HLA-A2 specific antibody) and surface levels of MHC-I were quantified by flow cytometry upon gating on eGFP positive cells. (C) Nef-V_C or empty backbone (pcDNA3.1) and eGFP were co-transfected and surface stained with W6/32 MHC-I antibody and surface levels of MHC-I were quantified by flow cytometry after upon on GFP positive cells. Histograms are representative of 3 independent experiments.

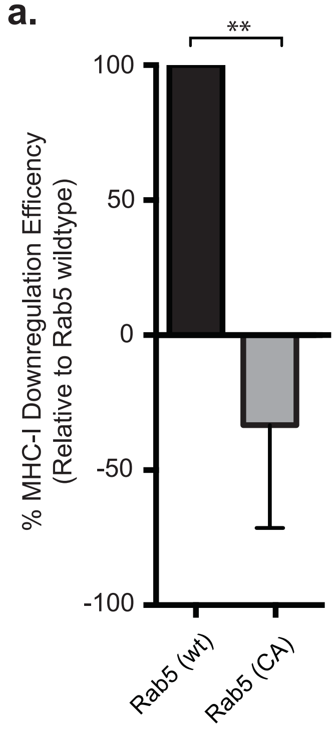

Supplemental Figure S2: Disruption of early endosomal regulation interferes with Nef-mediated MHC-I downregulation.
(A) HeLa cells were co-transfected with mCherry-tagged Rab5-constitutively active (CA) or Rab5 and Nef-eGFP or empty eGFP encoding backbone. Twenty-four hours post transfection, cell surface levels of MHC-I were measured by flow cytometry by gating on GFP and mCherry positive cells. Downregulation efficiency was calculated relative to Rab5 (wt) using the following formula: Relative MFI = { [1-$Rab5_{mut}Nef/Rab5_{mut}\Delta Nef$)] / [1-($Rab5_{wt}Nef/Rab5_{wt}\Delta Nef$)] } x 100. (B) A representative histogram of MHC-I surface levels from 3 independent experiments is shown. Error bars were calculated from 3 independent experiments (** indicates p-value < 0.01).

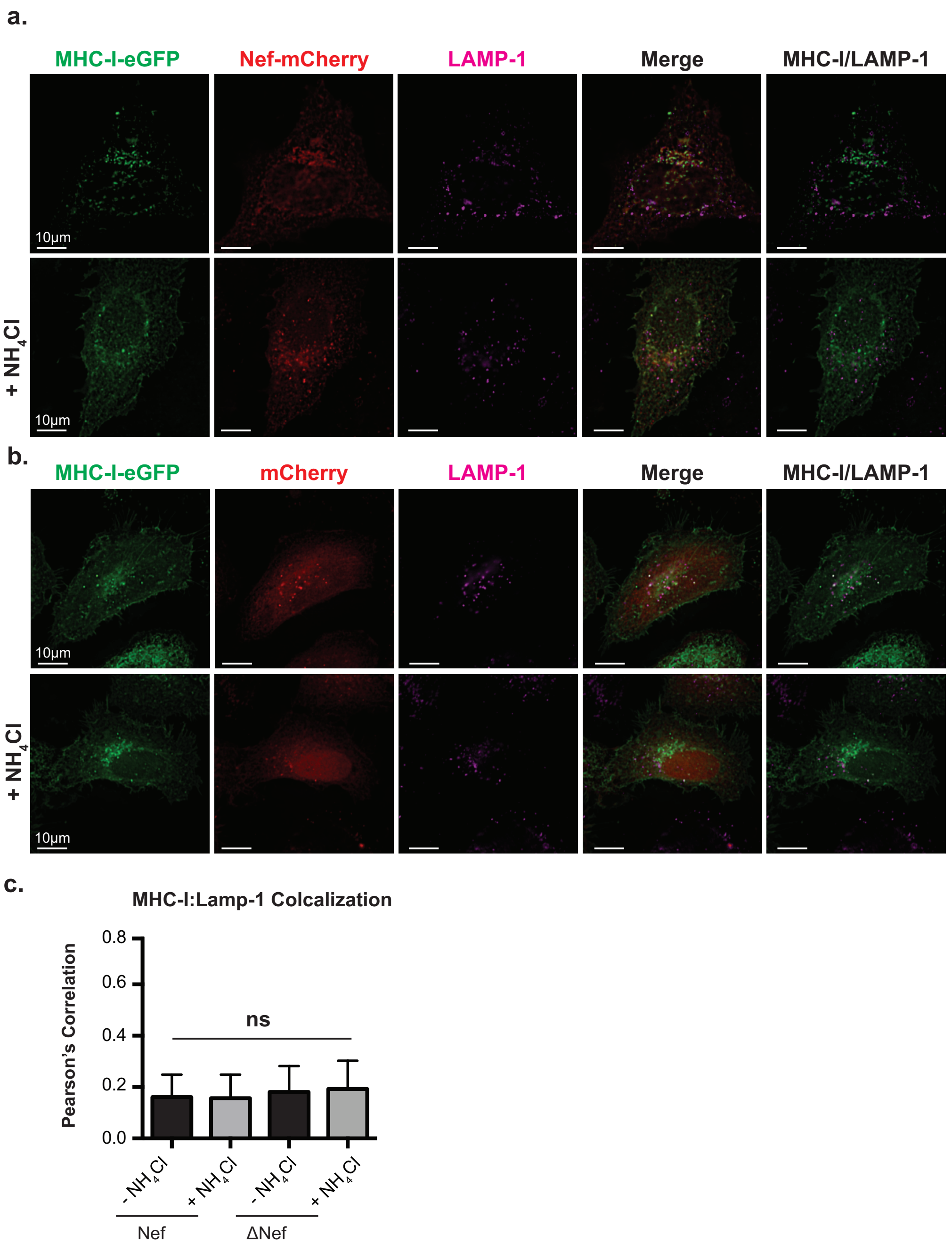

Supplemental Figure S3: Lysosomal localization of MHC-I-eGFP
(A) HeLa cells were transfected with MHC-I-eGFP (green) and Nef-mCherry (red). Twenty hours post transfection cells were treated with 100mM ammonium chloride for 4 hours. Following treatment, cells were fixed and immunostained for LAMP-1 (magenta). (B) HeLa cells were transfected with MHC-I-eGFP (green) and mCherry backbone (red). 20 hours post transfection, cells were treated with 100mM Ammonium chloride for 4 hours. Following treatment, cells were fixed and immunostained for LAMP-1 (magenta). (C) Co-localization of MHC-I and LAMP-1 were quantified by the Pearson's correlation through the JaCoP Plug-in on ImageJ. Error bars were calculated by quantification of at least 30 cells between 3 independent experiments, (ns: not significant).
